# Supplementary material for: A machine learning approach to predict in vivo skin growth
Source: Sci Rep. 2024 Jul 29;14:17456. doi: 10.1038/s41598-024-67056-z (PMC11286771; doi:10.1038/s41598-024-67056-z)
Supplement: Supplementary file 1 — Supplementary Information. [file 41598_2024_67056_MOESM1_ESM.pdf]

Supplementary Information for “A machine learning approach to predict *in vivo* skin growth”

Matt Nagle*, Hannah Conroy Broderick, Adrian Buganza Tepole, Michael Fop*† and Aisling Ní Annaidh*†

*Corresponding author(s). E-mail(s): matt.nagle@ucdconnect.ie; michael.fop@ucd.ie; aisling.niannaidh@ucd.ie;

Contributing authors: hannah.conroybroderick@ucd.ie; abuganza@purdue.edu;

†These authors contributed equally to this work.

A Two-Dimensional Finite Element Modelling

Initially, for computational efficiency, we developed a two-dimensional FE simulation consisting of a uniaxial pre-stretch on a block of skin followed by an impact on the surface; see Figure 1. It is presented here for its interpretability. The applied perturbation generates a wave that propagates along the surface of the skin. The vertical displacement of the node located 4 mm from the impact site was stored for analysis. This simulation makes the assumption that, for any small patch of skin, the complex stress distribution caused by the geometry of the expander can be approximated by a uniaxial stretch. To promote skin growth, the block of skin was stretched 18% past the natural pre-stretch value. This value was chosen as it is typical of the deformations seen in porcine skin at the apex of the expander after inflation (Han et al. Acta Biomaterialia 137, 136–146 (2022)). Due to the uniaxial stretch, both the stress and the growth fields will be homogeneous in the two-dimensional skin block.

To implement the FE model, the nonlinear FE package Abaqus/Standard (Dassault Systems, Waltham, MA) was used to statically pre-stretch the skin

using a displacement boundary condition and hold it in the stretched position to allow time for skin growth. Subsequently, Abaqus/Explicit (Dassault Systems, Waltham, MA) was used to dynamically perform the wave propagation procedure during which a 0.1 MPa pressure was applied for 2×10^{-5} s. As shown in Figure 1, the unstretched skin block had dimensions 10 mm x 10 mm, which was selected to minimise wave reflections interfering with the 4 mm waveform. The skin block was discretised into 80,000 CPE3 elements with 40,401 nodes.

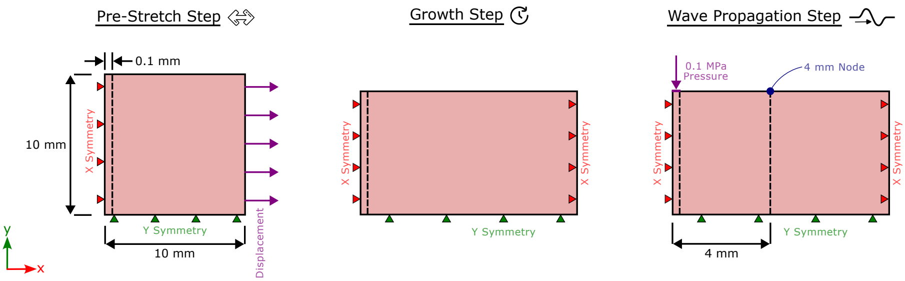

Fig. 1: Dimensions and boundary conditions for the two-dimensional FE model. (a) The uniaxial pre-stretch is generated using displacement boundary conditions. (b) The skin is held in this stretched configuration to allow time for skin growth. (c) The wave is generated by a 0.1 MPa pressure applied for 2×10^{-5} s. The vertical displacement of the surface node at 4 mm was stored for analysis.

As discussed in detail in the main text, a user-defined hyperelastic material was used to simulate the skin growth through tissue expansion process. In order to guarantee good coverage of the input space with a comparatively small number of samples, a Latin hypercube sampling method was used to generate 1,000 unique sets of material parameters (μ, k, θ_{nat}, and ρ) using the parameter ranges in Table 1. For each of these "subjects", a two-dimensional FE simulation was performed for 4 scenarios:

1. The skin was statically stretched to the natural pre-stretch value and the dynamic wave propagation procedure was performed. The displacement-time curve of the 4 mm surface node was stored for analysis (measurement 1).
2. The skin was statically stretched 18% past the natural pre-stretch value, held in that stretched configuration for 10^{-5} days (< 1 second), and the dynamic wave propagation procedure was performed. The displacement-time curve of the 4 mm surface node was stored for analysis (measurement 2).
3. The skin was statically stretched 18% past the natural pre-stretch value, held in that stretched configuration for 3.5 days, and the dynamic wave

propagation procedure was performed. The displacement-time curve of the 4 mm surface node was stored for analysis (measurement 3).

4. The skin was statically stretched 18% past the natural pre-stretch value, held in that stretched configuration for 7 days. The resulting growth of the skin was stored for analysis.

Material Property	Range	Units
μ	$[0.01667, 0.1]$	MPa
k	$[0.2, 1.92]$	day^{-1}
θ_{nat}	$[1.05, 1.2]$	
ρ	$[1060.2, 1171.8]$	$\text{kg}\,\text{m}^{-3}$

Table 1: Material property ranges used for the input space.

As discussed in the main text, our goal is to use the data from the three wave propagation scenarios to predict the growth at the 7-day check-in period, along with the subject-specific material properties such as the growth rate of the skin (k), the natural pre-stretch (θ_{nat}), and the stiffness of the material (μ).

B Two-Dimensional FE Results

As detailed in Section A, in each two-dimensional simulation, a uniaxial static pre-stretch was used to simulate the deformation past the natural pre-stretch caused by an inflating skin expander or similar deformation. The skin is held in this pre-stretched state, allowing it to grow. Over time, as the skin grows, the stress in the skin decreases; see Figure 2. This process can be expressed as the transition between elastic deformation to irreversible deformation, corresponding to skin growth. When the skin is stretched past the natural pre-stretch, skin growth is triggered, causing the elastic deformation to decay back towards the natural pre-stretch value; see Figure 3. Notably, the increased dimensions of the stress-free state at the end of the simulation underscore the irreversible skin growth; see Figure 2.

After the static pre-stretch and growth, a wave was propagated through the skin surface, see Section A. The peak and trough of the surface wave as well as the stress distribution can be seen in Figures 4 (a) – (c) and the resulting 4 mm waveform can be seen in Figure 4 (d).

As outlined in the main text, the primary goal of this study was to use non-invasive measurements from a wave propagation procedure to predict skin growth and other material properties of interest. Specifically, an ANN was trained to take the baseline, 0 day, and 3.5 day waveforms (consisting of the normal displacement of a node on the surface of the skin as a function of time) as input values. For the two-dimensional FE data, the target variables were the homogeneous growth value at day 7, the shear modulus μ, the growth rate k, and the natural pre-stretch θ_{nat}.

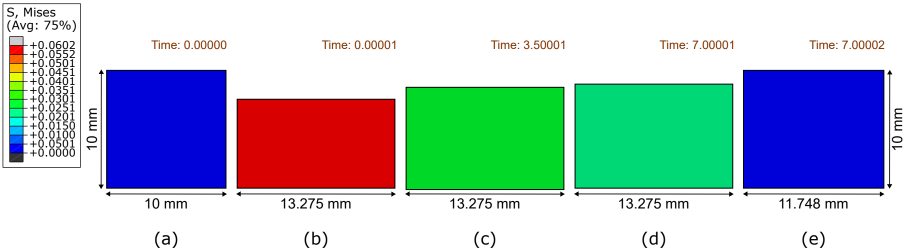

Fig. 2: Evolution of the Von Mises stress (MPa) throughout the 2D FE simulation. (a) The stress-free skin block at the start of the simulation, (b) the stress immediately after the uniaxial stretch 18% past the natural pre-stretch of the skin, (c) the stress after 3.5 days of growth, (d) the stress after 7 days of growth and (e) the stress-free skin block at the end of the simulation, indicating the extent of permanent tissue growth. Neo-Hookean material with a shear modulus $\mu = 0.058335$ MPa, a density $\rho = 1120\,\mathrm{kg\,m^{-3}}$, a growth rate $k = 1.2\,\mathrm{day}^{-1}$ and a natural pre-stretch $\theta_{\mathrm{nat}} = 1.125$ (12.5% extension).

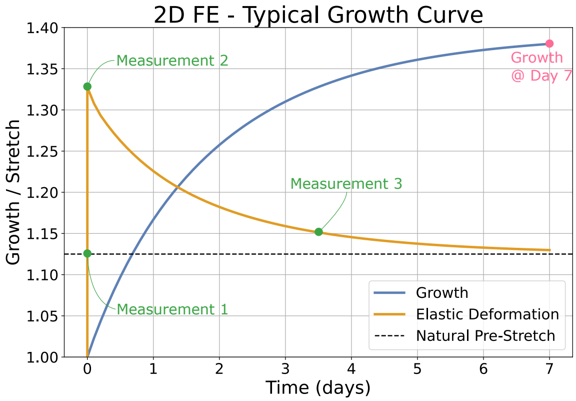

Fig. 3: Growth and elastic deformation of the skin as a function of time. Note that the elastic deformation 18% past the natural pre-stretch occurs on day 0, where the growth is 0 and the elastic deformation is at its maximum. The skin is held in this stretched state for 7 days, during which the elastic deformation decays towards the natural pre-stretch as the skin grows. Time points where the wave propagation measurements were taken (the ML inputs) are marked in green, and the growth at 7 days (the ML output) is marked in pink. Neo-Hookean material with a shear modulus $\mu = 0.058335$ MPa, a density $\rho = 1120\,\mathrm{kg\,m^{-3}}$, a growth rate $k = 1.2\,\mathrm{day}^{-1}$ and a natural pre-stretch $\theta_{\mathrm{nat}} = 1.125$ (12.5% extension).

The architecture of the ANN was tuned using a 10-fold cross-validation (CV) procedure with 5 repeats to ensure robustness and account for random variability in the predictive performance. As a performance metric, the R^2 value

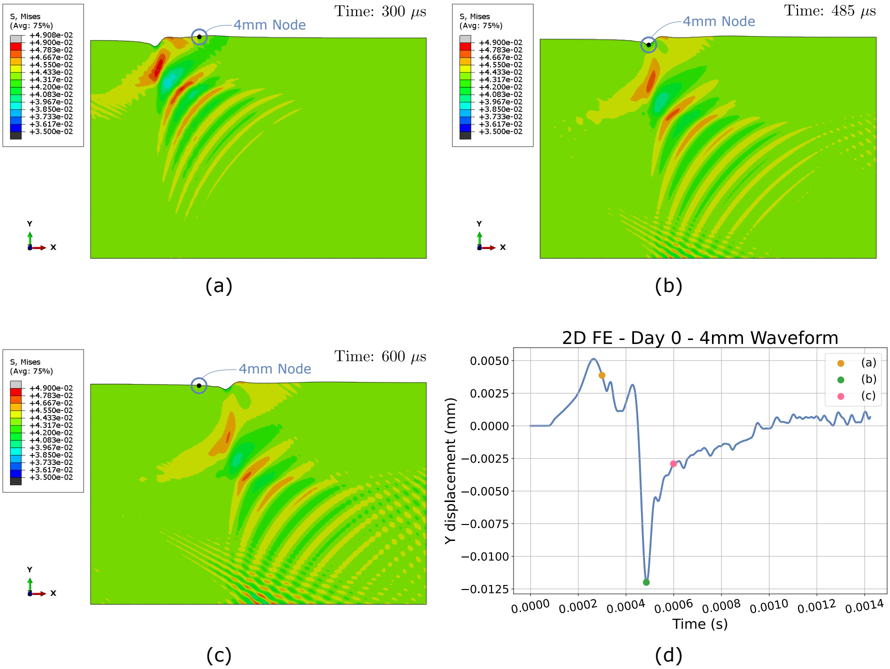

Fig. 4: (a-c) Von Mises stress (Pa) at different time points in the deformed neo-Hookean material. Note that the surface node 4 mm away from the impact is highlighted in black. (d) The resulting 4 mm waveform, highlighting the three time points shown in panels (a), (b) and (c). Shear modulus $\mu = 0.058335$ MPa, a density $\rho = 1120\,\text{kg}\,\text{m}^{-3}$, a growth rate $k = 1.2\,\text{day}^{-1}$ and a natural pre-stretch $\theta_{\text{nat}} = 1.125$ (12.5% extension). Note, the y-displacement has been scaled by a factor of 20 for easy visualisation of the surface wave.

computed between the ANN estimated output and the FE simulation output for each of the 4 target variables was calculated on the unseen test set subjects. The distribution of the performance for each of the target variables across various hidden layer architectures is depicted in Figure 5.

Out of the hidden layer architectures tested, it was found that three fully connected sequential hidden layers with 8, 16 and 8 nodes, respectively, yielded the best performance. The median performance metric is reported in Table 2. As discussed in the main text, to obtain a visual indication of the ANN predictive performance, we can examine a specific 90%/10% train-test split; see Figure 6. These results indicate that the ML model exhibits remarkably accurate predictions of the growth and the material properties of interest using non-invasive measurements obtained from the wave propagation procedure.

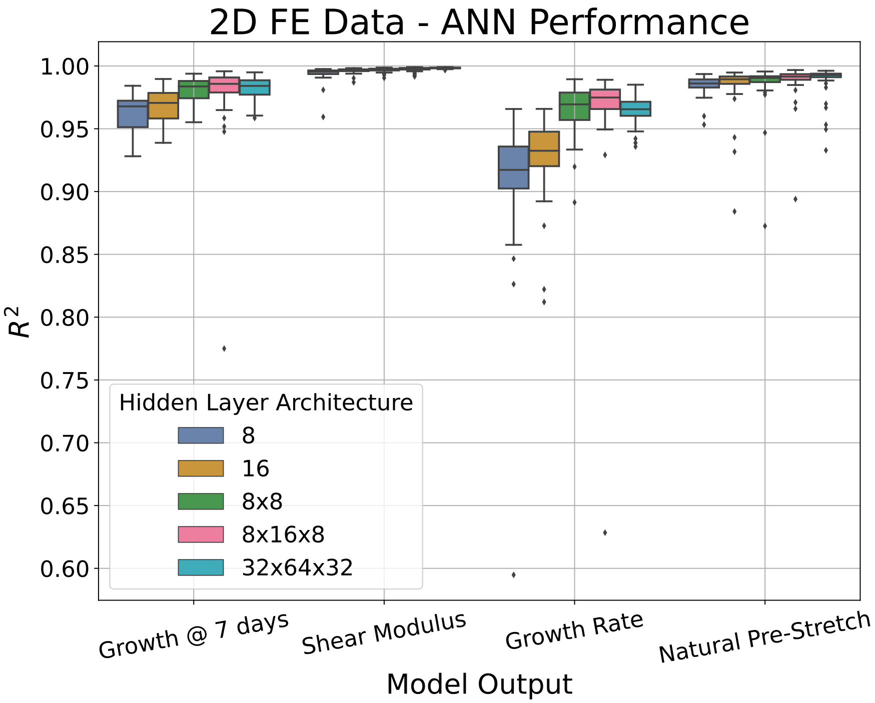

Fig. 5: Distribution of the performance resulting from the 10-fold CV procedure with 5 repeats, measured using the R^2 value for various hidden layer architectures for the four target variables: homogeneous growth value at day 7, shear modulus μ, growth rate k, and natural pre-stretch θ_{nat}. Out of the architectures tested, the $8 \times 16 \times 8$ node hidden layer architecture was found to have the best performance.

Target Variable	Median R^2 from 10-fold CV	Standard Deviation of R^2 from 10-fold CV
Growth at 7 days (θ_g)	0.9857	0.0313
Shear Modulus (μ)	0.9981	0.0018
Growth Rate (k)	0.9749	0.0499
Natural Pre-Stretch (θ_{nat})	0.9916	0.0148

Table 2: Median R^2 and standard deviation of R^2 from the 10-fold CV procedure with 5 repeats for the ANN trained on the 2D FE data with hidden layer architecture consisting of 8, 16 and 8 nodes.

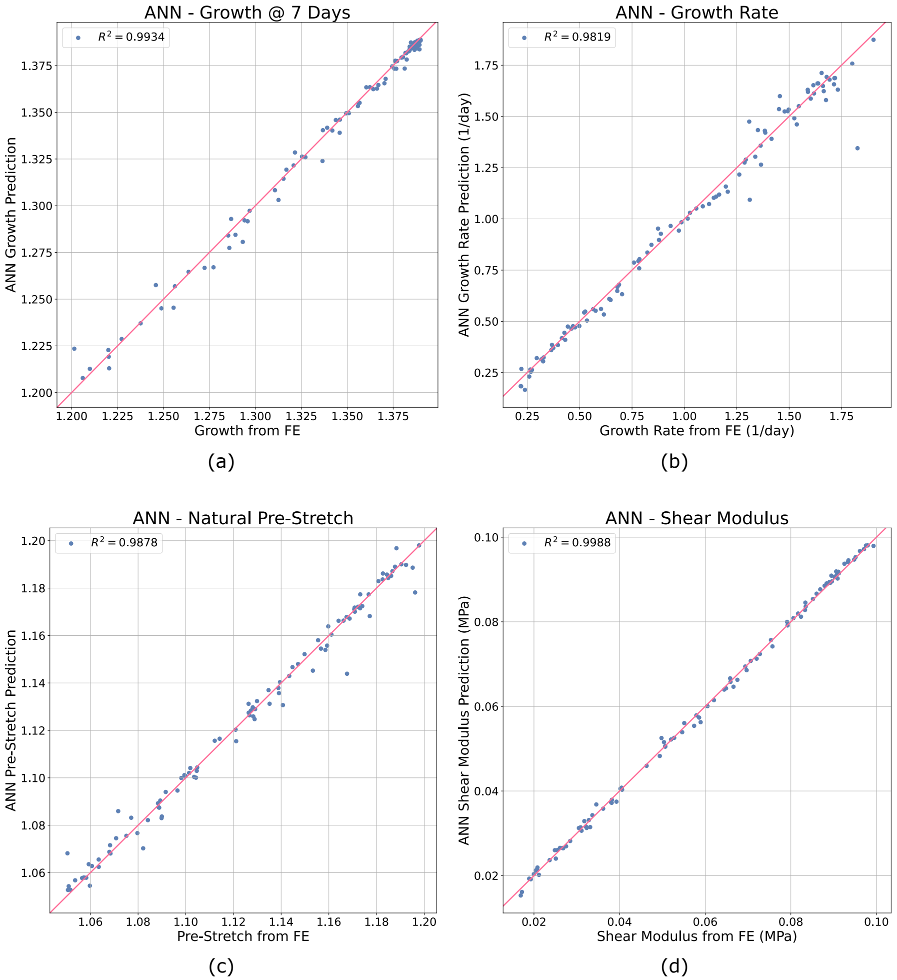

Fig. 6: Performance of the ANN model trained on 90% of the dataset and tested on the remaining unseen 10%. For each data point, the x coordinate is the "true" value of the target variable extracted from the FE simulation and the corresponding y coordinate is the ANN prediction of the target variable given the baseline, 0 day, and 3.5 day waveforms for that subject. As shown, the ANN has extremely high predictive accuracy.

C Data Processing

As discussed in the main text, prior to model training, the waveforms from the FE simulation were interpolated onto a regular grid of 1,001 equally spaced values between 0 and 0.0125 seconds. This was necessary due to the variation in the length and sampling rate of the waveforms extracted from the FE simulations for subjects with different material properties (and therefore wave speeds). Note that 1,001 values were chosen simply as a convenient number that samples a value every 1.25×10^{-5} s. This sampling rate was selected as it was enough values to capture the variation in the waveforms but also resulted in a manageable number of inputs for efficient ANN training. The sampling rate, if chosen poorly, could affect the algorithm's prediction accuracy. For example, if too many values are chosen (greatly increasing the dimensionality of the input space), it may become difficult or costly for the ANN to converge on an optimal set of coefficients during training. Conversely, if too few values are chosen, variation in the waveforms will be lost, making it difficult for the ANN to identify differences between the waveforms and therefore reducing prediction accuracy.

The data processing procedure is as follows (see Figure 7): First, the surface node closest to 5 mm away from the impact site is identified and the x, y and z-displacement curves are stored and used to construct the waveform normal to the surface. Then, a one-dimensional spline of degree 4 is fit to the normal waveform data and used to interpolate the waveform onto a regular grid of 1,001 equally spaced values between 0 and 0.0125 seconds. Finally, a training dataset is constructed where each virtual subject is listed along with their corresponding unique skin material properties, growth at 7 days and the baseline, day 0 and day 3.5 waveforms.

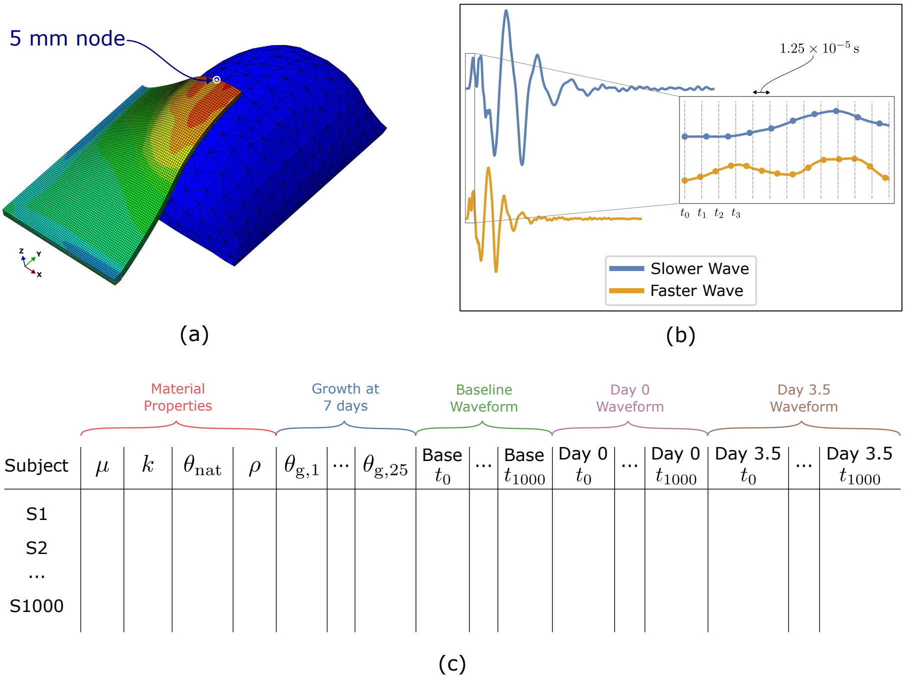

Fig. 7: Visualisation of the key data processing steps prior to model training. (a) The surface node closest to 5 mm away from the impact site is identified and the displacement normal to the skin's surface is constructed and stored. (b) Note that for different subjects with different skin material properties (and therefore wave speeds) the length and sampling rate of the waveforms extracted from the FE simulations will differ. After the interpolation step, all waveforms consist of 1,001 equally spaced values between 0 and 0.0125 seconds (i.e. will be evaluated at $t_0, t_1, ..., t_{1000}$). (c) The normal displacement at each regular time interval is stored alongside the virtual subject's unique skin properties and growth at 7 days (which, for the three-dimensional FE simulation, consists of 25 growth field values). Note that this process is performed for the baseline, day 0 and day 3.5 waveforms for every subject to create the dataset used for model training.
